# Adaptation of the sexual and reproductive empowerment scale for adolescents and young adults in Kenya

**DOI:** 10.1371/journal.pgph.0001978

**Published:** 2023-10-26

**Authors:** Elizabeth K. Harrington, Ouma Congo, Syovata Kimanthi, Annabell Dollah, Maricianah Onono, Nelly Mugo, Ruanne V. Barnabas, Elizabeth A. Bukusi, Ushma D. Upadhyay

**Affiliations:** 1 Department of Obstetrics & Gynecology, University of Washington, Seattle, Washington, United States of America; 2 Department of Global Health, University of Washington, Seattle, Washington, United States of America; 3 Center for Microbiology Research, Kenya Medical Research Institute, Nairobi, Kenya; 4 Center for Clinical Research, Kenya Medical Research Institute, Nairobi, Kenya; 5 UW-Kenya, Nairobi, Kenya; 6 Division of Infectious Disease, Massachusetts General Hospital, Boston, Massachusetts, United States of America; 7 Department of Obstetrics, Gynecology, and Reproductive Sciences, University of California, San Francisco, San Francisco, California, United States of America; Tata Institute of Social Sciences, INDIA

## Abstract

Measuring empowerment is critical to understanding the level of control adolescents and young adults (AYA) have over their sexual and reproductive health (SRH) behaviors, and could provide a key window into addressing their unique SRH needs. We adapted the Sexual and Reproductive Empowerment (SRE) scale for AYA for use in an East African context. This multi-method qualitative study sampled 15–23 year-old female adolescents and young adults in Kisumu, Kenya. We conducted in-depth interviews (n = 30) and analyzed transcripts with an inductive, constant comparison approach. Empowerment domains were integrated with Kabeer’s (1999) framework in a conceptual model, which we referenced to revise the original and develop new scale items. Items underwent expert review, and were condensed and translated through team-based consensus-building. We evaluated content validity in cognitive interviews (n = 25), during which item phrasing and word choice were revised to generate an adapted SRE scale. Participants (n = 55) had a median age of 18 (range 16–23), and 75% were under 19 years. We categorize three types of adaptations to the SRE scale: new item generation, item revision, and translation/linguistic considerations. We developed nine new items reflecting AYA’s experiences and new domains of empowerment that emerged from the data; new domains relate to self-efficacy in accessing sexual and reproductive health care, and how material needs are met. All items were revised and translated to echo concepts and language relevant to participants, navigating the multilingualism common in many African countries. Centering the voices of female Kenyan AYA, this study provides insight into measuring the latent construct of adolescent sexual and reproductive empowerment in an East African setting, and supports the adapted SRE scale’s content validity for Kenya. We detail our multi-method, theory-driven approach, contributing to limited methods guidance for measure adaptation across contexts and among diverse adolescent populations.

## Introduction

While sexual and reproductive health (SRH) rights and needs of adolescent and young adults (AYA) in sub-Saharan Africa are increasingly acknowledged and prioritized, they continue to experience entrenched SRH disparities [1, 2]. Against the backdrop of gender inequality, female youth experience disproportionate risks of sexual coercion and assault, HIV and other sexually-transmitted infection (STI) acquisition, and unintended pregnancy [3–5]. AYA face multiple layers of barriers to sexual and reproductive wellbeing, ranging from unequal power in sexual relationships, to a lack of SRH knowledge and financial resources, to confidentiality concerns and bias in health care settings [6–8]. While many AYA are able to overcome these barriers to SRH and wellbeing, exercising agency and choice in their SRH care-seeking and behaviors, many others do not [9]. Divergent levels of sexual and reproductive empowerment may help explain differential SRH care-seeking, outcomes, and why programs such as those promoting contraceptive access and HIV prevention often have uneven effects among African youth [10, 11].

There has been renewed attention to conceptualizing and measuring women’s empowerment [12–14], but until recently, there were no measures of sexual and reproductive empowerment specific to adolescents and youth. The Sexual and Reproductive Empowerment Scale for Adolescents and Young Adults (SRE scale) [15] is a new multidimensional measure validated in a diverse, representative population of adolescents and youth aged 15–24 in the United States (S1 Table). Comprised of 23 items in 7 subscales, the SRE scale demonstrated high internal consistency and reliability in psychometric evaluation; scores were significantly associated with indicators for access to SRH information and access to SRH services. Furthermore, for every 1-point increase in the full SRE scale, female participants had a 6% higher odds of using their desired contraceptive method [15]. The SRE scale has not yet been adapted or validated for a non-US setting.

Researchers and implementers routinely use measures that were developed and psychometrically tested in another geographic and/or sociocultural setting [16]. It is also common to adapt a measure for a new setting, often in a different language, without clearly defining the methods that were used to adapt the measure. While these practices may be reasonable—and necessary for feasibility—in some cases, it is essential to consider how the content validity of a transplanted measure may be altered [16]. While there are a variety of high-quality resources for scale development [17, 18], considerably less guidance is available regarding methodology for scale adaptation across diverse contexts, especially for populations of youth. The guidelines published by Beaton *et al*. provide recommendations for a five-stage process of “cross-cultural adaptation” [19]. These guidelines, which focus on translation and cross-cultural equivalence, engage research participants towards the end of the process during item pre-testing. However, engaging the community of interest through in-depth qualitative research as the first step of adaptation may have key advantages.

Given the lack of methodological guidance in the literature regarding scale adaptation, this study outlines a conceptual and practical team-based process of measure adaptation for diverse populations. We identify key domains of sexual and reproductive empowerment relevant to the lived realities and reproductive choices of Kenyan female AYA, and describe how these findings were used to adapt the SRE scale for an East African context.

## Materials and methods

### Study design and sampling

**Fig 1:**
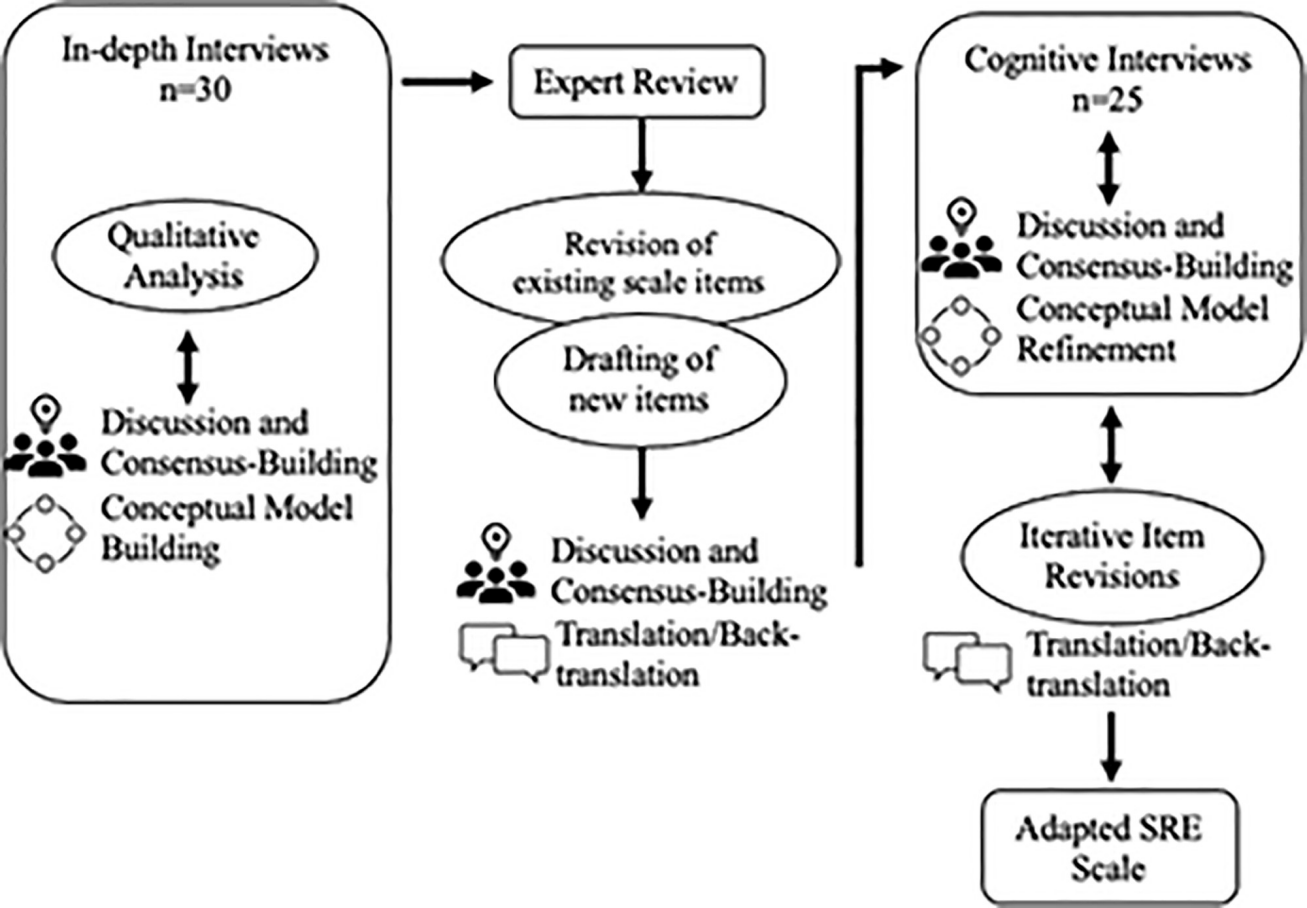
Methods flow diagram.

This multimethod qualitative study [20] incorporated in-depth interviews (IDIs), expert review, and cognitive interviews (CIs) (Fig 1). Interviews were conducted between May-September 2021 in urban and peri-urban Kisumu, which is Kenya’s third-largest city and primarily inhabited by the Luo ethnic group. This setting was chosen due to existing Kenya Medical Research Institute (KEMRI) research infrastructure and co-authors’ extensive regional experience. For both the IDIs and the CIs, the research team used purposive maximal variation sampling [21] to recruit 15–23 year-old cisgender female AYA who were sexually active with a male partner in the last year; spoke English, Dholuo, or Kiswahili; and had the capacity for pregnancy (not currently pregnant or sterilized). Participants were recruited from community-based venues, such as markets and youth gathering places near schools and colleges, with the assistance of community health volunteers, and an ongoing clinical trial cohort [22] in equal numbers. Individuals who participated in IDIs were not eligible to participate in CIs. We oversampled adolescents 18 and younger to optimize the representation of their perspectives in light of this group’s considerable underrepresentation in SRH research due to the inherent challenges of engaging minors in research [23, 24].

### Data collection and analysis

#### Theoretical framework

We drew on Kabeer’s foundational theoretical perspectives on women’s empowerment, which define empowerment as both process and outcome: “the expansion of people’s ability to make strategic life choices in a context where this ability was previously denied them” [25]. This conceptualization of empowerment underlies many empowerment measures [26–28], and is particularly apt for AYA, whose social, cognitive, and physical development is also in process along with their autonomy to make life choices. Kabeer describes the three “interrelated dimensions” of empowerment as resources (the material and social environment), agency (“the ability to define one’s goals and act upon them”), and achievements (valued outcomes achieved from resources and agency).

#### In-depth interviews and conceptual model-building

Data collection started with semi-structured IDIs (n = 30), which conceptually explored female AYA’s sense of power over their sexual and reproductive experiences, including sexual relationships, contraceptive choices and use, pregnancy, and abortion (S1 File). Most interviews were conducted in a combination of languages per participants’ preference (English, Dholuo, and Kiswahili); audio-recordings were transcribed and translated into English by the interviewers themselves. The coding team used a constant comparison approach to qualitative analysis, drawing on Grounded Theory methods with concurrent data collection and analysis [29]. We developed a codebook combining *a priori* concepts of interest (drawn from our group’s prior research and ongoing experience engaging AYA in research in the region) and empowerment domains with raw data-inspired codes. The transcripts were triple-coded in Dedoose (Version 8.0.35) with regular team meetings to reach consensus on divergent code application and guide analytic summaries.

**Fig 2:**
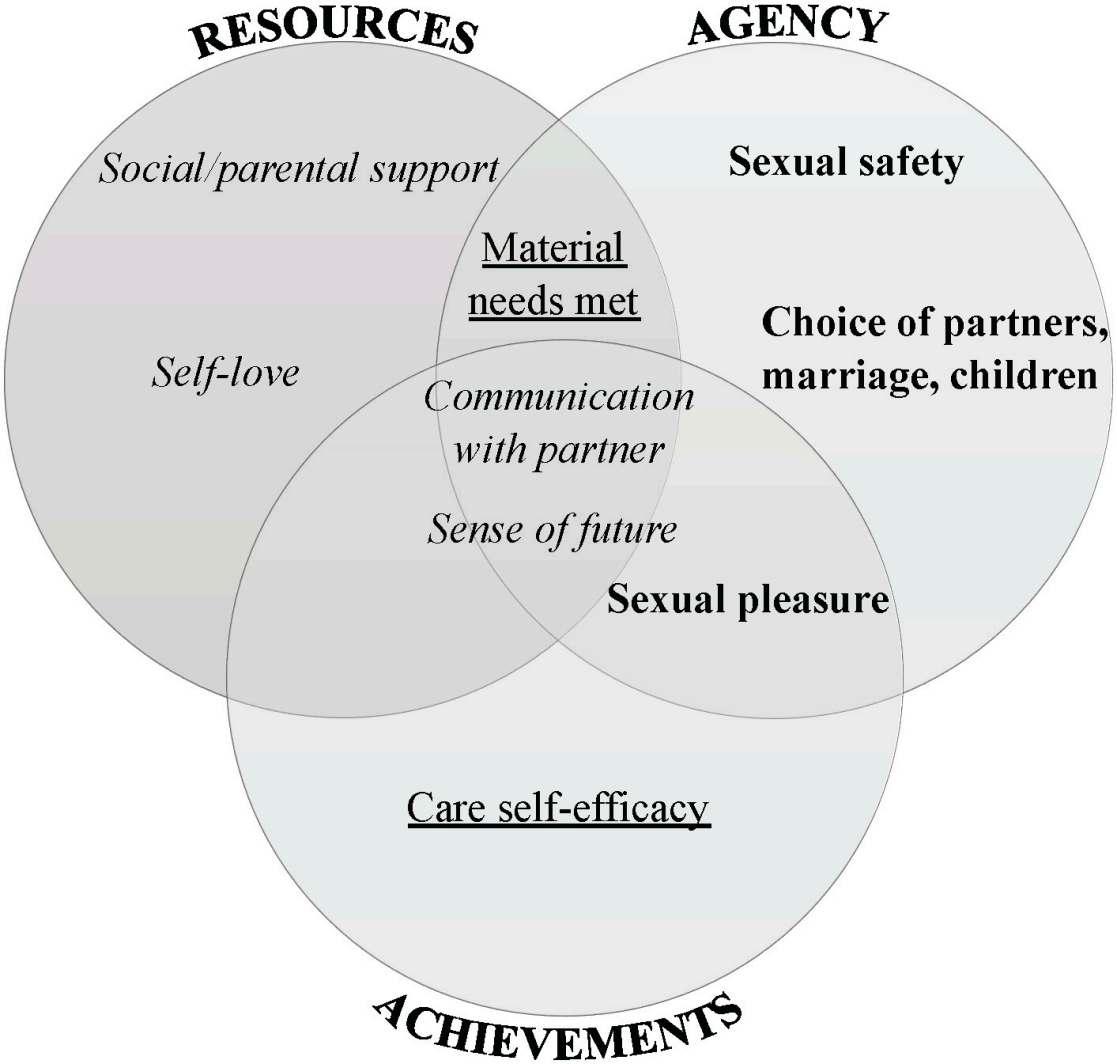
Conceptual model of sexual and reproductive empowerment for adolescents and young adults (Kenya)*. *Italicized font specifies an original SRE scale domain that did not conceptually change. **Bold** font specifies an original SRE scale domain to which new items were added. Underlined font specifies an emergent domain.

We integrated existing SRE domains with Kabeer’s resources-agency-achievements framework [25] in a conceptual model (Fig 2), which we then referenced to develop new scale items. The three dimensions of Kabeer’s framework are highly interrelated, and we used a Venn diagram to illustrate how the new domains informed by our qualitative findings (emergent domains; underlined), and original SRE scale domains (bold font and italicized) fit conceptually into the framework. We generated new scale items to represent the emergent domains and added new scale items to existing domains where participants’ lived experience provided additional insights into original domains.

#### Item development and expert review

We drafted 21 new scale items in order to better reflect the lived experiences of participants and incorporate the emergent empowerment domains. Through a process of team-based iterative consensus-building, including the entire analytic team, we condensed the new items and revised the existing items for clarity. All items were then translated into Kiswahili and Dholuo and backtranslated into English by different experts for comparison. The response choices use the original 5-point Likert scale of agreement: not at all true (0), a little true (1), moderately true (2), very true (3), and extremely true (4); a higher score indicates a higher level of empowerment. Some new items were written to be reverse-coded, meaning that “not at all true” would be scored at 4 and “extremely true” at 0.

The adapted scale (English) then was reviewed by three Kenyan experts and researchers in adolescent sexual and reproductive health research, policy, behavioral science, and implementation science. Their feedback was used to guide further revisions in preparation for CIs.

#### Cognitive interviews

The adapted scale was evaluated in CIs (total n = 25) to assess face validity. Interviewers administered the adapted SRE Scale items, then used a structured interview guide to explore comprehension, relevance, and linguistic appropriateness of each item. Interviewers took detailed field notes on participants’ responses during interviews, and selectively transcribed excerpts of the audio-recordings to add direct participant quotations to their field notes. Field notes were discussed at weekly team meetings and item phrasing and word choice were iteratively revised in real time, with additional translation input from contributors external to the study team. During this process, more than half of the new items were dropped based on performance in CIs.

### Research team and reflexivity

The qualitative interviewers who collected all study data were cisgender women, trilingual, and had 5–10 years of qualitative data collection and transcription/translation experience. Study team training included values clarification on adolescent sexuality and contraceptive use, and a team culture was established that privileged the horizontal exchange of ideas. The members of the coding team (EKH, OC, SK) have experience providing SRH care for AYA. The primary author has over ten years of experience collaborating on qualitative research in Kenya. All authors have been engaged in collaborative reproductive health research in the study region or have expertise specific to adolescent sexual and reproductive empowerment. For further details, please refer to our structured reflexivity statement [30] in S5 File.

### Ethics statement

This study was approved by the Kenya Medical Research Institute (KEMRI) Scientific Ethics Review Unit (P00152/4193), the Kenya National Commission for Science, Technology, and Innovation (NACOSTI/P/21/10896), and the University of Washington Human Subjects Division (STUDY001172). A waiver of parental consent for minors was granted by the above institutions, as it was determined during ethical review that study procedures themselves (a one-on-one qualitative interview) posed minimal risk to adolescents, and the primary potential risk associated with participation was invasion of privacy and social harm [23], which could occur with inclusion of parents. The inclusion criteria for the study, which included prior sexual activity, were not disclosed outside of the study team or during recruitment. Formal written consent (18 years and older) and assent (15–17 year-olds) was obtained. The consent and assent materials clearly explained the sensitive nature of the content that would be discussed in the interviews, and potential participants were asked to answer comprehension questions at the end of the process that emphasized the voluntariness of participation and the possibility of psychosocial discomfort during interviews as well as the small risk of a breach of confidentiality. The assent process encouraged adolescents to consult with a trusted adult about participation in the study, if they felt comfortable doing so. Authors EKH, OC, SK, and AD had access to identifiable information about participants during and after data collection.

## Results

IDI and CI participants (n = 55) ranged in age from 16–23, with a median age of 18 (IQR 17–19.5). The majority (69%) were currently students, and most (61%) were attending secondary school. Most participants (91%) were currently romantically partnered, had been sexually active in the last month (60%), and had never been pregnant (69%) (Table 1).

**Table 1:** Participant characteristics.

Characteristic, total n = 55	n (%)	median(IQR)
Age, years		18 (17–19.5)
Age group, years		
15–19	41 (75)	
20–23	14 (25)	
Educational achievement		
Primary school or less	7 (13)	
Secondary school incomplete	25 (45)	
Secondary complete or above	23 (42)	
Currently a student	38 (69)	
Currently partnered	50 (91)	
Age of primary partner, years (n = 50)		23 (19–29)
Source of financial support		
* Self*	11 (20)	
* Parent/guardian*	43 (78)	
* Romantic partner*	30 (55)	
Employment status		
No employment	43 (78)	
Formal sector employment	3 (5)	
Informal sector employment	9 (16)	
Age at sexual debut		16 (14–17)
Ever contraceptive use	50 (91)	
Current contraceptive use	36 (65)	
Nulligravid	38 (69)	
Religion		
Protestant Christian	30 (55)	
Catholic Christian	18 (33)	
Muslim	2 (4)	
Traditional religions	4 (7)	
Other	1 (2)	

In the following results, we outline the types of adaptations we made to the SRE scale grouped into three primary categories: new item generation, item revision, and translation and linguistic considerations. We illustrate how changes to the SRE scale were driven by our data, providing specific examples of qualitative findings used in the adaptation process that resulted in an adapted SRE scale with 23 original and nine new items (Table 2) in three languages.

**Table 2:** New Adapted SRE scale items and domains.

Domain	New item
Choice of partners, marriage, children	I would try to choose a romantic or sexual partner who would help me achieve my life goals.
	If my partner provided me with things I need, I would feel like I should have sex.*
Material needs met	
Sexual safety	I would be able to say no to sex if I do not want to have sex.
	I have had to do sexual things to please someone when I didn’t want to.*
Sexual pleasure	I am afraid of having sex.*
Care self-efficacy	I am confident that I could get the services I need to prevent a pregnancy or an infection.
	I would not worry about others judging me if I have decided to use a method to prevent pregnancy.
	What other people think about methods to prevent pregnancy is less important than what I think and want.
	I feel confident that I could get condoms if I wanted to use them.

*Designed to be reverse-coded

### New item generation (In-depth interviews)

#### Domain: Sexual pleasure

Sexual pleasure, a domain and subscale of the original SRE scale, emerged as a multifaceted domain relevant to Kenyan AYA. While several participants described sexual relationships where their pleasure was prioritized by themselves and male partners, the majority did not feel they could initiate sexual interest and felt anxiety about being branded a certain kind of girl who likes sex: *“‘cause other boys can say maybe this girl is just on sex so I can fear even to tell that I want sex”*(IDI 17). Furthermore, participants frequently framed sexual desire and the anticipation of sexual pleasure as dangerous. Desire for sexual pleasure could lead one to give in to condomless sex; many linked sexual pleasure with risk of the catastrophic (for them) consequences of unintended pregnancy and HIV. Several participants described fearing having sex, or even talking about sex, due to prior coercive experiences or health consequences. They talked about the need to control sexual urge, which one participant (IDI 16) named “the ‘I don’t care’ trait.” A 17 year-old participant (IDI 26), who was not currently sexually active, described her strategy of using oral contraceptives to intentionally quell her sexual desires:

*[I use pills] [t]o control myself from sex…I am taking them [pills] so that they can help me*. *So that I can control myself…so that it can reduce my urge for sex*. *I saw my friends used it to help themselves*, *so when I came to* [clinic name] *I decided to request for them*.

This theme of fear of sex and sexual pleasure and their consequences as hazardous to one’s health and life, inspired the new item, **I am afraid of sex**, to capture this negative aspect of sexual pleasure.

#### Domains: Material needs met, choice of partners

Sex with a transactional or exchange component was highly normalized among participants, who shared a range of personal and peer narratives. Most commonly, participants described receiving gifts or cash from boyfriends, who then expected sex. Several others reported the necessity of having sex with an older, often married, man in order to have their school fees paid or basic needs met. An 18-year-old secondary school student (IDI 4) explained:

*Yeah*, *you know something about boyfriends or young men*, *they believe that for a relationship to be stable*, *you have to cut out competition*. *So…they will always prefer to buy you gifts and all that*. *So you know when your boyfriend…does that for you*, *he wants something in return and you are not going to buy him gifts in return*. *So you will feel bad because you are using all his money and you have to return*, *and in return it is always sex*.

Balancing material needs and sexual partnerships emerged as a pervasive factor in participants’ understanding of their sexual and reproductive power. A 19-year-old mother with a young daughter (IDI 24) narrates her perspective on depending on an older man for financial support:

*He has more power than I do because he caters for all my needs*, *such that if I made a mess then all that is gone*. *I have no time hurling insults at him*, *even when he wrongs me because if I do*, *he will stay quiet and not talk to me*. *I will instead be forced to go and beg him to talk to me because after all it’s me who is in need*.

This salient theme was conceptualized as *material needs met*, a domain of sexual and reproductive empowerment that is highly related to *choice of partners*. A new item developed to measure this domain is: **If my partner provided me with things I need, I would feel like I should have sex**.

#### Domain: Sexual safety

The domain of sexual safety in the original SRE scale reflects one’s sense of bodily autonomy and safety. Interviews explored AYA’s sense of physical safety in their daily activities, as well as dynamics around consent within sexual relationships. Participants frequently cited personal experiences with sexual coercion and assault, from nuanced gendered expectations around sexual roles to experiences of sexual assault by a peer or stranger. AYA spoke of complex sexual expectations related to financial support, fear of male partner violence, and the desire to avoid accusations of infidelity and conflicts with their male partners if they said no to sex. This participant, a 17 year-old nulligravid secondary school student with a 19 year-old boyfriend (IDI 20) describes some situations where she has insisted on saying no to her boyfriend’s sexual advances, but other times feels so much pressure she gives in:

*Then another thing that brings about a disagreement is sometimes when I am in my periods*, *he wants me to have sex with him and if I refuse*, *he starts saying that those are just my usual tricks and [men] are used to them*, *that I just want to avoid him to go and sleep with another boy outside*. *So this normally creates violence*.

Given that the item reflecting coercive sex in the original SRE scale (“I do not feel afraid that I will be forced to do something sexually when I do not want to”) uses the concept of being “forced,” which may not apply to the very wide range of coercive experiences participants described, we developed new items to better represent more subtle sexual coercion. For example: **I have had to do sexual things to please someone when I didn’t want to**.

#### Domain: Care self-efficacy

The ability of AYA to overcome societal and health systems barriers to accessing sexual and reproductive health services, such as contraception and abortion, was conceptualized as care self-efficacy. Participants described several stigmatizing narratives surrounding contraceptive use among youth that circulated in their communities. Many believed that contraceptive methods were unhealthy for young women, and threatened their fertility if they had never given birth. As a 19 year-old participant (IDI 22) who had recently made the decision with her boyfriend to stop using injectable contraception explained, “…*someone in the hospital I went to told me that Depo can destroy the eggs in your womb*. *It totally destroys all the eggs in your womb such that you are unable to get kids in future*. *That is why I hate it*.*”* Another source of stigma was the concept that contraception invited sexual promiscuity; for example, that using a method to prevent pregnancy would lead to indiscriminate sexual activity with multiple partners due to a lack of pregnancy risk. Concern about lack of privacy and male partner discovery of contraceptive use were frequently cited. Several adolescents, including this 16 year-old participant (IDI 7), reported “obeying’ their partners when it was demanded that they discontinue a method.

*I did not tell him that I was going to get a family planning method*. *He later reprimanded me saying that why did I go for family planning*, *and that did it mean that I did not trust him…he also said that it meant that I had other side partners and that was why I had gone for a family planning method…I told him*, *that was not true and that family planning simply prevents one from getting pregnant*. *But he refused*, *and that made me to go and remove it*. *He actually made sure that he escorted me while I was going to remove it*.

An example of an item added within the new care self-efficacy domain is: **What other people think about methods to prevent pregnancy is less important than what I think and want**.

### Item revision (Cognitive interviews)

Prior to initiating CIs, we used iterative, team-based discussion and consensus-building to revise the wording of the original 23 SRE scale items for improved comprehension in the Kenyan context. In many instances, Kenya-based team members recommended that the English phrasing be modified for simplicity and word choice. For example, in the item “Walking down the street, I feel like by body is my own,” we replaced “street” with “road,” as the latter word is more familiar in Kenya and also creates a mental image that is more consistent with norms in both urban and rural settings.

Several items were revised in response to the CIs (see Table 3). For example, in the first few interviews, participants had difficulty understanding the item “My sexual needs and desires are important,” as the concept of a sexual “need” was unfamiliar. The item was revised to “What I want sexually is important;” however, several participants answered “not at all true” to the item, interpreting it as “Sex is a priority for me.” An 18 year-old student who was in a sexual relationship (CI 9) and using condoms intermittently stated, “*for now it [sex] is not that much important but it will be very important in the future…currently I fear the outcome of sex*. *I might decide to have sex and then I end up getting pregnant*.*”* The item was further revised to “My sexual desires are important,” which prompted participants to think more about the balance between their and their partners’ sexual desires. In the new item, “I would not worry about others calling me promiscuous if I have decided to use a method to prevent pregnancy,” several participants had difficulty understanding the word “promiscuous.” A 19-year-old secondary school graduate who lives in a informal settlement where she runs a grocery kiosk (CI 18) suggested using the word “judging” instead: “I would not worry about others judging me….” She recalled a recent experience where she used emergency contraception twice in a month, despite feeling that her friends looked down on her and tried to discourage her.

**Table 3:** Item revision examples.

Domain	Item Pre-Cognitive Interview	Revised Item
*Original SRE Scale Items*		
Comfort talking with partner	If I had a romantic partner, I would feel comfortable voicing disagreements with them.	If I had a romantic or sexual partner, I would feel comfortable telling them I disagreed with them.
Choice of partners, marriage, children	I have the power to control if and when I have children.	I have the power to decide if and when I have children.
Sexual safety	Walking down the street, I feel like my body is my own.	Walking down the road, I feel like my body is my own.
Self-love	I am worthy of love.	I deserve to be loved.
*New Items*		
Care self-efficacy	I would not worry about others calling me promiscuous if I have decided to use a method to prevent pregnancy.	I would not worry about others judging me if I have decided to use a method to prevent pregnancy.
Material needs met; Choice of partners, marriage, children	I would feel like I should have sex if my partner helped me financially.	If my partner provided me with things I need, I would feel like I should have sex.
Sexual safety	I usually feel safe.	Dropped

The cognitive interviewing process also confirmed that many original and new SRE scale items were indeed relevant to Kenyan youth. For example, the item “I know my body well” prompted participants to talk about knowing their bodies in a variety of ways, including knowing one’s HIV status, health needs, and how to experience sexual pleasure. A 19-year-old adolescent (CI 7) put it this way: “*I know myself*. *I do know myself like I know when something strange is happening to my body…Personally*, *when I am just about to have my periods*, *I really crave for sex with my boyfriend…”* Furthermore, participants comprehended and often deeply connected with items related to the self-love domain, in particular the item “I like myself,” which was revised to “I love myself” based on AYA feedback. Participants were often effusive; field notes describe one 17 year-old participant’s expression “brightening” when asked about the “I love myself” item: *“I just love myself the way I am*. *I am just proud of myself*, *the way I am*, *the way I have been created*, *my size*: *yes*, *I am just okay as I am”* (CI 8).

### Translation and linguistic considerations

As in many African settings, multiple languages are spoken in western Kenya. Adaptation of the SRE Scale for relevance in western Kenya required translation into two widely-spoken regional languages: Kiswahili, a bantu language widely spoken throughout Kenya and East Africa, and Dholuo, a Nilotic regional language spoken primarily by the Luo ethnic group (See S2 Table for translated items). The iterative process of translation and backtranslation revealed differences in connotation of sensitive words, which were flagged in track changes by expert translators and brought to the team for discussion. A key example of this process was translation of the items that included the word “sex” into Dholuo. One translator translated the word sex as “nindruok,” and the backtranslated version retained meaning. Another translator raised concern that to some, the word “nindruok” would be perceived as a vulgar term, and suggested “terruok” as an alternative. A tie-breaker translator confirmed that “terruok” was most appropriate.

CIs were conducted in all three languages, which added to the complexity of understanding how item phrasing, word choice, and comprehension altered cognitive processes. Three participants who chose Dholuo in their CIs shared that the item “Walking down the road, I feel like my body is my own” made them think of the “My dress, my choice” campaign in Kenya. This motto was coined during feminist protests after several instances of women being publicly stripped by groups of men after wearing short skirts in Nairobi and Mombasa [31]. The item was well-understood by most participants in all three languages, but appeared to prompt slightly different cognitive associations in the different languages. Finally, multilingualism and language-mixing presented dynamic challenges to cognitive interviewing and administration of the SRE scale in one language. In Kenya, as in many settings in sub-Saharan Africa, using two or more languages in the same sentence is typical, and verbally administering the SRE scale in one language often used artificial phrasing and words that were less well-known to some participants.

## Discussion

We describe a detailed methodological approach to adapting the SRE scale for use among female adolescents and youth in Kenya, and provide adapted scale items for use and further study in sub-Saharan African contexts. This research contributes to two distinct gaps in the literature. First, this paper adds to the limited guidance for researchers who desire to adapt measures for new settings. Second, we present domains of sexual and reproductive empowerment relevant to female AYA in Kenya, integrating them into a theory-driven conceptual model based on Kabeer’s resources-agency-achievements framework [25].

In this study, IDIs yielded key insight into existing SRE scale domains, and revealed additional, context-relevant themes and perspectives around young women’s sexual and reproductive experiences and autonomy. While a rigorous qualitative analysis may seem impractical for some measure adaptation projects, this step may be particularly critical when social norms vary greatly between settings, when working with adolescents or other groups who are underrepresented in research, and when the latent construct is sensitive or stigmatized. Specifically relating to the latter, IDIs allow for rapport-building with inductive exploration of participant narratives and experiences [32]. Concepts or domains that are “missing” from the original measure will not necessarily come up in structured CIs, which may contribute to poor representation of already marginalized adolescent populations in research. For example, our qualitative interview findings prompted exploration of the new “care self-efficacy” domain and the development of four novel items. This domain takes into account dominant social norms and stigma [33] around young women’s sexuality and contraceptive use, including beliefs about contraception that are prevalent in Kenya and elsewhere in sub-Saharan Africa [34, 35],

The process of categorizing the measurement domains within Kabeer’s empowerment model helped elucidate the overlapping nature of resources, agency, and achievements in sexual and reproductive empowerment among youth. For example, using Kabeer’s framework, we conceptualized sexual pleasure as both an “achievement” and demonstrating “agency” among participants. Sexual pleasure and desire are underexplored dimensions of adolescent SRH [36, 37], especially in sub-Saharan African contexts, limiting insight into adolescents’ sexual and reproductive empowerment. Reminiscent of themes in Tolman’s (1994) landmark work on adolescent sexual desire, our findings exposed “common threads of fear and joy, pleasure and danger” [38] interwoven in participant narratives of their sexual experiences. Many participants highlighted the fear and danger they related to sexual desire, and the practice of using oral contraceptives for the main purpose of reducing desire, to which we were unable to find other references in the literature, is a case in point. Future research is needed to examine how adolescent SRH programming could more effectively affirm exploration of sexual desire and pleasure while promoting positive health outcomes.

Consistent with prior studies [6, 8, 39, 40], power in AYA sexual relationships (including the ability to negotiate health-related behaviors like condom use when desired, or escape intimate partner violence) was influenced by gendered and social norms, as well as participants’ needs for money and other material goods. Adolescents and young women described nuanced forms of exchange sex, often but not always in parallel with love or desire, as the norm among themselves and their peers. Our findings and inclusion of a new item directly addressing how partner financial support or gift-giving relates to sexual behaviors and choice of partners is also affirmed by the HIV prevention literature. Sex in exchange for money or gifts among youth has been widely studied in a variety of sub-Saharan African settings as a common social practice as well as a risk factor for HIV acquisition [41–43]. While we decided to conceptualize the adapted item relating to exchange sex such that answering in the affirmative would indicate less empowerment, our group also discussed how using sex to get what you want or need could be perceived as agentic. However, within the contexts and narratives of this study’s participants, feeling compelled to have sex as a result of financial support or gifts was overall portrayed as disempowering.

Finally, while the results in this manuscript are organized around the adaptations we made to the SRE scale, the items representing several original SRE scale domains were altered very little: these include social/parental support, communication with partner, self-love, and sense of future. Our analytic team encountered many examples of positive self-image, self-love and auspicious visions for their futures; we reflected that research in global SRH rarely portrays adolescent girls and young women as agents with these characteristics. Ongoing work should emphasize and support such positively-framed elements of sexual and reproductive empowerment to change the dominant narrative that female AYA, particularly in low- and middle-income countries, are collectively disempowered.

Our adaptation approach and results have several strengths, including the multidisciplinary team, community-based recruitment, the triangulation of multiple qualitative methods, and use of a theory-driven conceptual model to guide measure adaptation. This study also has important limitations. We collected data in one Kenyan county, and the findings may not apply to other social, political, and linguistic contexts. However, we found that themes from our data were germane to the HIV prevention and adolescent SRH literatures in ways that support cross-context parallels in young African women’s power in the sexual and reproductive domain [6, 41, 43–45]. While the methodological rigor our binational team brought to this work is a strength, we recognize that engaging a community advisory board of AYA would have further strengthened our approach by introducing additional participatory opportunities to center the perspectives of AYA. Another limitation of our study is lack of diversity in gender and sexual orientation. The original SRE scale was designed to be inclusive of female, male, and trans/nonbinary youth. This study focused exclusively on cisgender female youth. Future work should include boys, men, and trans/nonbinary individuals, as well as those with diverse sexual orientations, in adaptation efforts.

To support the sexual and reproductive health and autonomy of adolescents and youth globally, it is essential to understand their level of power over sexual and reproductive behaviors and decisions [8, 40]. This multimethod qualitative study demonstrates appropriate content validity of the adapted SRE scale in western Kenya. Additional research is needed to evaluate the psychometric properties and overall performance of the adapted version of the original 23 items and the nine new items. Psychometric analysis could guide item reduction for a more parsimonious scale, assess the underlying factor structure of the adapted scale, and determine if the adapted SRE scale is associated with a relevant outcome (construct validation) [17]. With adapted, context-relevant measures, researchers and implementers will be able to better understand how SRH needs, preferences, access, and outcomes differ by level of empowerment, enabling them to develop more person-centered interventions and innovations in service delivery.

## Supporting information

S1 Table Original sexual and reproductive empowerment for adolescents and young adults scale items with administration and scoring instructions. (DOCX)

S2 Table Revised and translated adapted SRE scale items with instructions. (DOCX)

S1 File In-depth interview guide, English version. (PDF)

S2 File University of Washington Human Subjects Division approval. (PDF)

S3 File Kenya Medical Research Institute Scientific Ethics Review Unit approval. (PDF)

S4 File PLOS inclusivity in global research questionnaire. (DOCX)

S5 File Structured reflexivity statement. (PDF)
