# Supplementary material for: Adaptation of the sexual and reproductive empowerment scale for adolescents and young adults in Kenya
Source: PLOS Glob Public Health. 2023 Oct 26;3(10):e0001978. doi: 10.1371/journal.pgph.0001978 (PMC10602344; doi:10.1371/journal.pgph.0001978)
Supplement: S1 Table — (DOCX) [file pgph.0001978.s001.docx]

| **Domain (Subscale)** | **Item** |
| --- | --- |
| Comfort talking with partner | If I had a romantic partner, I would feel comfortable talking about whether or not I want to have children with them. |
|  | If I had a sexual partner, I would feel comfortable telling that person if I wanted to use a method to protect against infection or pregnancy, even when they did not want to |
|  | If I had a romantic partner, I would feel comfortable voicing disagreements with them. |
| Choice of partners, marriage, children | I can freely choose if I get married. |
|  | I can freely choose who I marry. |
|  | I have the power to control if and when I have children. |
| Parental support | I have a parent or guardian who would help me with my problems and troubles if I needed. |
|  | I have a parent or guardian who accepts me as I am. |
|  | I have a parent or guardian who trusts me to make the right decisions. |
|  | I have a parent or guardian who helps me achieve my goals in life. |
| Sexual safety | I am able to do the things I want to do without worrying about my safety. |
|  | Walking down the street, I feel like my body is my own. |
|  | I do not feel afraid that I will be forced to do something sexually when I do not want to. |
|  | I feel safe in my current living situation. |
| Self-love | I like myself. |
|  | I am worthy of love. |
|  | I know my body well. |
|  | My body belongs to me. |
| Sense of future | I can imagine what my future will be like. |
|  | I have an idea of how I can eventually reach my goals. |
| Sexual pleasure | My sexual needs or desires are important. |
|  | I think it would be important to focus on my own pleasure as well as my partner's during sexual experiences. |
|  | I expect to enjoy sex. |

Supplemental Table 1: Original Sexual and Reproductive Empowerment for Adolescents and Young Adults Scale Items with Administration and Scoring Instructions

| **Instructions** | **Answer Choices** |
| --- | --- |
| **Instructions:** Each of the following statements may or may not apply to you. Please rate how true each statement is for you.  *Note: You don't need to have ever had sex or currently have a sexual partner to answer this survey. If you are unsure about how to answer any items, please give your best guess.* | Answer choices:   - Not at all true - A little true - Moderately true - Very true - Extremely true |

**Scoring Instructions**

For each of the 23 SRE scale items, assign a value from 0 (Not at all true) to 4 (Extremely true) based on participant response. The score for each of the 7 subscales is calculated as the sum of the items in the subscale. If there are any missing item responses within a subscale, the weighted sum should be calculated using the following formula:

*Weighted subscale score = actual subscale sum * subscale items / non-missing subscale items*

For example, the subscale Comfort talking with partner contains 3 subscale items, allowing for a range of subscale score from 0 to 12. If a participant answered only 2 of the 3 items in that subscale (e.g. 3/Very true for both items) and left one missing, the actual subscale sum of the 2 items (6) would be multiplied by the number of total subscale items (3) then divided by the number of non-missing subscale items (2) for a final weighted subscale score of 9.

The overall SRE scale score is calculated as the sum of each subscale score, or the sum of all 23 scale items. If any of the 23 scale items are missing, the weighted total scale score is calculated in the same manner as for the weighted subscale scores.
