# Supplementary material for: Adaptation of the sexual and reproductive empowerment scale for adolescents and young adults in Kenya
Source: PLOS Glob Public Health. 2023 Oct 26;3(10):e0001978. doi: 10.1371/journal.pgph.0001978 (PMC10602344; doi:10.1371/journal.pgph.0001978)
Supplement: S2 Table — (DOCX) [file pgph.0001978.s002.docx]

S2 Table: Revised and Translated Adapted SRE Scale Items with Instructions

| **Item Pre-Translation** | **Language** | **Translated Item** |
| --- | --- | --- |
| **Revised Original SRE Scale Items (23)** | | |
| If I had a sexual partner, I would feel comfortable talking about whether or not I want to have children with them. | Kiswahili | Ikiwa ningekuwa na mpenzi wa ngono, ningehisi vyema kuzungumzia ikiwa ninataka kuwa na watoto naye au la. |
|  | Dholuo | Ka an kod Jahera, anyalo winjo yot wacho ka adwaro bedo kod nyathi kode kata ok adwar. |
| If I had a sexual partner, I would feel comfortable telling that person if I wanted to use a method to prevent infection or pregnancy, even when they didn’t want to. | Kiswahili | Ikiwa ningekuwa na mpenzi wa ngono, ningejihisi vyema kumwambia mtu huyo ikiwa ningetaka kutumia njia ya kuzuia maambukizo au mimba, hata wakati hawakutaka. |
|  | Dholuo | Ka an kod Jahera, anyalo winjo yot nyise kaponi adwaro tiyo kod yo mar gengo tuo kod ich kata ka ok adwar. |
| If I had a romantic or sexual partner, I would feel comfortable telling them I disagreed with them. | Kiswahili | Ikiwa ningekuwa na mpenzi wa kimapenzi au mpenzi wa ngono, ningejihisi vyema kuwaambia sikubaliani nao. |
|  | Dholuo | Kaponi an kod Jahera kata jalno ma aterora godo, anyalo winjo yot nyisogi ka ok wawinjre e wach. |
| I can choose if I want to get married or not. | Kiswahili | Ninaweza kuchagua ikiwa ninataka kuolewa au la. |
|  | Dholuo | Anyalo yiero ka adwaro donjo e keny kata ooyo. |
| I can choose who I get married to. | Kiswahili | Ninaweza kuchagua ni nani atakaenioa. |
|  | Dholuo | Anyalo yiero ng’atno ma adonjo godo e keny. |
| I have the power to decide if and when I have children. | Kiswahili | Nina uwezo wa kuamua ikiwa nitapata watoto na ni wakati gani. |
|  | Dholuo | An gi teko mar yiero ka adwaro nyithindo kata kinde ma adwaro bedo kod nyinthindo. |
| I have a parent/guardian who would help me with my problems if I needed. | Kiswahili | Nina mzazi / mlezi ambaye angenisaidia kwenye shida zangu nikiwahitaji. |
|  | Dholuo | An kod Janyuol/Jarit manyalo konya loyo chandruogena ka adwaro. |
| I have a parent/guardian who accepts me the way I am. | Kiswahili | Nina mzazi / mlezi ambaye hunikubali vile nilivyo. |
|  | Dholuo | An kod Janyuol/Jarit ma orwaka kaka an. |
| I have a parent/guardian who trusts me to make the right decisions. | Kiswahili | Nina mzazi / mlezi ambaye huniamini kuwa nitafanya maamuzi bora. |
|  | Dholuo | An kod Janyuol/Jarit ma nitie kod geno kuoma mondo atim yiero mabeyo. |
| I have a parent/guardian who helps me achieve my goals in life. | Kiswahili | Nina mzazi / mlezi ambaye hunisaidia kufikisha malengo yangu maishani. |
|  | Dholuo | An kod janyuol/jarit makonya chopo dwarona mag ngima. |
| I am able to do the things I want to do without worrying about my safety. | Kiswahili | Nina uwezo wa kufanya mambo ninayotaka kufanya bila kuwa na wasiwasi juu ya usalama wangu. |
|  | Dholuo | Anyalo timo gigo madwaro timo maonge parruok kuom arita mara. |
| Walking down the road, I feel like my body is my own. | Kiswahili | Nikitembea barabarani, nahisi mwili wangu ni wangu binafsi. |
|  | Dholuo | Ka awuotho e yo, awinjo ka denda en mara awuon. |
| I do not feel afraid that I will be forced to do something sexually when I don’t want to. | Kiswahili | Sihisi uoga kwamba nitalazimishwa kufanya tendo lolote la kingono wakati sitaki. |
|  | Dholuo | Ok awinj ka aluor ni ibiro chuna timo gimoro ma otenoro kod terruok ka ok adwar |
| I feel safe in my current living situation. | Kiswahili | Ninajihisi usalama kwa hali yangu ya sasa ya kuishi. |
|  | Dholuo | Awinjo ka an gi rit maber gi kaka adak sani. |
| I love myself. | Kiswahili | Ninajipenda. |
|  | Dholuo | Aherora awuon. |
| I deserve to be loved. | Kiswahili | Ninastahili kupendwa. |
|  | Dholuo | Awinjore mondo ohera. |
| I know my body well. | Kiswahili | Ninatambua mwili wangu vizuri. |
|  | Dholuo | Ang’eyo denda maber. |
| My body belongs to me. | Kiswahili | Mwili wangu ni mali yangu kibinafsi. |
|  | Dholuo | Denda en mara. |
| I can imagine what my future will be like. | Kiswahili | Ninaweza kufikiria jinsi maisha yangu ya baadaye yatakavyokuwa. |
|  | Dholuo | Anyalo temo paro kaka ngima na mabiro biro chalo. |
| I have an idea of how I can achieve my goals. | Kiswahili | Nina wazo jinsi ninaweza kufikia malengo yangu. |
|  | Dholuo | An gi paro ewi kaka anyalo chopo dwarona mag ngima. |
| My sexual desires are important. | Kiswahili | Ninacho tamani kingono ni cha muhimu. |
|  | Dholuo | Gombona ewi weche terruok duon’gna |
| I think it would be important to focus on my own pleasure as well as my partner's during sex. | Kiswahili | Ninadhani itakuwa muhimu kuzingatia raha yangu mwenyewe pamoja na ya mpenzi wangu wakati wa ngono |
|  | Dholuo | Aparo ni en gima duong keto pacha kuom mor mayudo kachiel kod mar jaherana e seche mag terruok. |
| I expect to enjoy sex. | Kiswahili | Natarajia kufurahia ngono. |
|  | Dholuo | Ageno mar winjo mit e seche mag terruok. |
| **Item Pre-Translation** | **Language** | **Translated Item** |
| **New SRE Scale-Kenya Items (9)** | | |
| I would not worry about others judging me if I have decided to use a method to prevent pregnancy. | Kiswahili | Siwezi jali ikiwa wengine watanihukumu ikiwa nimeamua kutumia njia ya kuzuia mimba. |
|  | Dholuo | Ok abidewo ka jomoko wuoyo ewiya ka ayiero mar tiyo kod yo mar gen’go ich. |
| What other people think about methods to prevent pregnancy is less important than what I think and want. | Kiswahili | Maoni ya watu wengine juu ya njia za kuzuia mimba sio muhimu kuliko kile ninachofikiria na ninachotaka. |
|  | Dholuo | Gima jomoko paro kuom yore mag gen’go ich ok duon’gna moloyo gima aparo kendo adwaro. |
| I feel confident that I could get condoms if I wanted to use them. | Kiswahili | Ninajiamini kuwa nitapata kondomu ikiwa nataka kuzitumia. |
|  | Dholuo | An gi adieri ni anyalo yudo rabo yunga ka adwaro tiyo kodgi. |
| I would be able to say no to sex if I do not want to have sex. | Kiswahili | Nitaweza kusema hapana kwa ngono ikiwa sitataka kufanya ngono. |
|  | Dholuo | Anyalo bedo kodnyalo mar wacho ooyo ne terruok ka ok adwa terora. |
| I have had to do sexual things to please someone when I didn’t want to. | Kiswahili | Nimelazimika kufanya matendo yeyote ya ngono ili kumpendeza mtu wakati sikutaka. |
|  | Dholuo | Asebedo kod achune mar timo gige terruok mondo amor ng’ato ka on ne adwar. |
| I am afraid of having sex. | Kiswahili | Ninaogopa kufanya ngono. |
|  | Dholuo | Aluoro terruok. |
| If my partner provided me with things I need, I would feel like I should have sex. | Kiswahili | Ikiwa mpenzi wangu ananipatia vitu ninavyohitaji, nitahisi ni lazima nifanye ngono. |
|  | Dholuo | Kajaherana miyaga gigo ma adwaro, anyalo winjo ni onego aterra kode. |
| I am confident that I could get the services I need to prevent a pregnancy or an infection. | Kiswahili | Nina uhakika kwamba naweza kupata huduma ninazohitaji kuzuia mimba au maambukizo. |
|  | Dholuo | An kod adieri ni anyalo yudo chenro ma adwaro tiyo godo e geng’o ich kata tuo. |
| I would try to choose a romantic or sexual partner who would help me achieve my life goals. | Kiswahili | Nitajaribu kuchagua mpenzi au mpenzi wa ngono ambaye atanisaidia kufikia malengo yangu ya kimaisha. |
|  | Dholuo | Anyalo temo yiero jahera kata osiep kisera manyalo konya chopo dwarona mag ngima. |

| **Language** | **Instructions** | **Answer Choices** |
| --- | --- | --- |
| English | **Instructions:** Each of the following statements may or may not apply to you. Please rate how true each statement is for you.  *Note: You don't need to have ever had sex or currently have a sexual partner to answer this survey. If you are unsure about how to answer any items, please give your best guess.* | Answer choices:   - Not at all true - A little true - Moderately true - Very true - Extremely true |
| Kiswahili | **Maagizo:** Kila mmoja ya taarifa zifuatazo zinaweza kukufaa au kutokukufaa. Tafadhali weka kwenye ratili jinsi kila taarifa ilivyo kweli kwako.  *Kumbuka: Sio lazima uwe umeshafanya ngono au uwe unayempenzi wa ngono ndipo uweze kujaza savei hii. Ikiwa hauna uhakika vile utajibu vitu vyovyote, tafadhali toa dhana yako.* | Jibu la chaguo:   - Sio kweli kabisa - Kweli kidogo - Kweli kiasi - Kweli sana - Kweli kabisa |
| Dholuo | Chike: Wach ka wach moluwogi nyalo kata ok nyal bedo ni winjore kodi. Kiyie to ler kaka wach ka wach en adiera ne in.  *Gik ma onego ing’e: Ok ochuno ni nyaka ibed ni iseterorie kata sani in kod jahera e ka idwok penjo gi. Ka ok in kod adieri kuom chiwo dwoko mora mora, kiyie chiw dwoko ma iparo ni owinjore.* | Dwoko ma ng’ato nyalo yiero   - Ok en adier chutho - Adier matin - Adier moromo - Adier ahinya - Adier moromo ahinya |
