# Supplementary material for: Adaptation of the sexual and reproductive empowerment scale for adolescents and young adults in Kenya
Source: PLOS Glob Public Health. 2023 Oct 26;3(10):e0001978. doi: 10.1371/journal.pgph.0001978 (PMC10602344; doi:10.1371/journal.pgph.0001978)
Supplement: S1 File — (PDF) [file pgph.0001978.s003.pdf]

MARA Study
In-depth interview guide – v1.2, 15 July 2021

Introduction to the study:
Thank you for taking the time to participate in this study. Our team is interested in understanding how young women make choices about their reproductive health. I will be asking you questions about your life, relationships, sexual experiences, and pregnancy and family planning experiences. While these topics are sometimes hard to talk about, I will not judge what you are telling me, and I appreciate your honest responses so that we can learn from you. If you do not feel comfortable answering a question, please let me know. Please keep in mind that there are no right or wrong answers to these questions. We will keep your identity private and from this point forward will use a number in place of your name. Your answers are completely private and no one will know your answers came from you.
Do you have any questions or concerns before we start the interview?

Parental and social support
First, I'd like to ask you some questions about the people who are important in your life.

Who would you say raised you?

If has/had two parents/guardians:
How would you describe your parents' relationship as you were growing up?

Please tell me about your living situation now.
Who do you live with?
Who provides financial support for you?

Who are the most important people in your life now? Why?

Relationships
Now, I'd like to ask you some questions about your romantic relationships. By romantic relationship, I am referring to a relationship where there are sexual feelings or you feel you are more than friends.

Are you currently in a romantic relationship?
What would you call this person? *E.g. boyfriend, husband, etc.*

If answers "no," ask if she has a current sexual partner. If yes, ask her to answer based on her current partner.

If does not have a current sexual partner, ask her to answer based on her last sexual relationship. Change questions to reflect past tense.

How long has the relationship been going on?
How serious would you say your relationship is? What makes it serious/not so serious?

Can you tell me about a time when you disagreed with your partner? What was the disagreement about? How did you and he deal with the situation?

Do you have any other current romantic relationships *(i.e. concurrent intimate partners)*? What about your partner?

When two people are in a romantic relationship, often there are topics that are talked about and also topics that tend to be avoided. Can you tell me about this in your relationship?

Do you and your partner talk about sex? How comfortable are you discussing sex with your partner? Why do you think this is?

Some people tell us they have more power than their partner, some say they have equal power as their partner, and some say that they have less power than their partner. What applies to you in your relationship? Why do you think that is?

Pregnancy
Now I'd like to ask you about your feelings about pregnancy.

If has been pregnant before: Can you tell me about your first pregnancy?
Probes:
- How did you feel about becoming pregnant? Did you want the pregnancy at that time?
    - *If unwanted or mistimed:* Please tell me more about the situation.
- How did your partner react to the pregnancy?
- How did your family react to the pregnancy?
- *For unwanted/mistimed pregnancies:* Were you using a method to prevent pregnancy at the time? Why or why not?

How would you feel if you found out you were pregnant today?
Probes:
- How do you think you would react? How would your partner react? How would your parents react?
- Why do you think you would react that way?
    - *For those who would react positively:* Is there any part of you that would be sad or upset about being pregnant? Could you explain that to me?
    - *For those who would react negatively*: Is there any part of you that would be happy or excited about being pregnant? Could you explain that to me?
- How do you think having a child now would affect your life? Why?

Can you tell me more about how you feel about having (more) children? When would be a good time in your life? Why would that be a good time?

How much control do you have over how many children you have and when you have them? Why/not?

Can you tell me about a time when you or someone you know became pregnant when they didn't want to be pregnant?
Probes:
- What are the options someone has if they become pregnant when they didn't want to be?
- What would *your* options be? How would you decide which option to choose?
- How much of a say would your partner have in the decision? What about your parents?
- Are there circumstances under which you think an abortion is the right choice? Which circumstances?
- Do you know where would you go if you or a friend needed an abortion? Where?

Pregnancy prevention
Now I have some questions about preventing pregnancy.

Could you tell me what the term family planning means to you?

How do you talk about family planning with your partner, if at all?
Probes:
- Who brings it up?
- When do you talk about it?
- *If no*, why do you think you don't talk about family planning?

What family planning or contraceptive methods have you used (if any)?
Probes:

- How did you decide to use that method?
- How did you decide to stop using that method?
    - How much did your partner/family weigh into the decision to use/stop using that method? How do you feel about how he/they were involved?

How does your partner feel about family planning? How do you know?
Probes:
- How does he/would he feel about you using family planning?
- Has he ever kept you from using family planning when you wanted to use it?

How would your parents respond if they found out you were using family planning? Why?

Choice of a particular family planning method is often based on women's personal preferences, in other words, the things they care most about.

When you think about choosing a family planning method, what comes to mind for you?

What are the most important factors for you when you think about choosing a family planning method? There is no right or wrong answer.
- *For example, some women want a method they can stop taking whenever they want, while others want a method that will work for years at a time like the implant in the arm. Other women want to minimize certain kinds of side effects, like irregular bleeding, or are most concerned about HIV prevention.*

Probes:
- How do you feel about using a method every time you have sex, like condoms or the E-pill? Why?
- Do you want a method that works for a period of time (like the injection or the implant), or would you prefer to have the option to stop it right away on your own (like the pill)? Why?
- How do you feel about irregular bleeding, or bleeding between periods caused by a method? How about if you knew it was normal and healthy to have that kind of bleeding while using the method? Why?
- How important are your friends' and family members' experiences with a method to you? Can you give an example of a story that affected how you feel about a particular family planning method?
- Have you ever used a method of family planning without your partner's knowledge? What were your reasons for using without him knowing? How important is it to be able to keep your method use secret?
- Which methods are easiest to keep private, in your opinion?
- How does (or would) cost affect your decision to use a method?
- How important to you is where you can get the method? For example, some people your age prefer to go to a clinic, others a pharmacy. Where would the ideal place to get a method be? Why is that?

I want you to imagine two girls your age. Both want to delay pregnancy for several years, and feel that getting pregnant now would be bad for their lives. They both live with their parents, who are not supportive of using a family planning method. They are both concerned about their ability to get pregnant in the future. One ends up deciding to use the injection and goes to the pharmacy to get it. The other uses condoms when her boyfriend will agree to use them, and eventually gets pregnant around your age. What do you think influenced these girls' different decisions? Why?

When it comes to using family planning, tell me about how that decision is made? What about which method to use?
Probes:
- Who has the most say about using a family planning method in your relationship? Who has the most say over choosing a particular method? Why do you think this is?
- How do you feel about your partner's level of involvement?
- How do you feel about your parents' involvement?

Sex and Coercion
Now I would like to ask you about your sexual experiences. Remember, no answer is wrong and everything you tell me is completely confidential.

In relationships sometimes both people want sex the same amount, and sometimes one partner wants sex more than the other: Can you describe what happens most of the time with your partner (or a past partner)?

Can you tell me about an experience when your sexual partner wanted to have sex when you didn't want to?
Probe: How did you feel about this? How often does this happen?

Do you feel or have you felt pressure to have sex? What usually ends up happening in these situations?

Could you tell me about your experience using condoms?
If has used condoms:
- How did you and your partner decide to use a condom? What was your main motivation in using the condom? What about your partner, do you think?
- Have you ever been in a situation where you wanted to use a condom and your partner didn't? What happened and how did you deal with that? What about a situation where your partner wanted to use a condom and you didn't?

If has never used condoms:
- Why do you think you have never used a condom? Whose decision was it to not use a condom?
- Has your partner ever refused to use a condom? Why was that? How did you deal with that?
- Do you have concerns about using condoms? What are your concerns?

It is common for young women to receive gifts and financial support from boyfriends. Sometimes young women feel pressure to have sex because they have a need for the money or the things provided by the boyfriend. Has this ever been true for you? Could you tell me more about the situation(s)?
Probes:
- *If said previously that partner provides financial support*: With your current partner, have you ever felt pressure to have sex because of gifts or money he provided for you? Could you tell me more about that?

Imagine you are walking alone down the street and pass a group of men on the corner with their motorbikes. How does that make you feel? Why? Can you describe an experience in a public space when you felt unsafe?

Life Goals
Now I am going to ask you some questions about your goals in life.

What do you see yourself doing in 5 years? What will your life be like?

Can you tell me what your partner thinks of these goals? (If they have not discussed them: What do you think he/she *would* think about these goals?)
What about your parents/guardians?

If currently unmarried: Can you tell me about how you feel about marriage?
Probes:
- Do you want to get married someday? Any idea when?
- How much say do your parents or other family members have in who you marry?

Thank you very much for participating in this study. Our team appreciates your time and your willingness to share this information. We will keep all your information private in the future. Do you have anything else you would like to tell me at this time? Any questions before we complete the interview?
