# Supplementary material for: Adaptation of the sexual and reproductive empowerment scale for adolescents and young adults in Kenya
Source: PLOS Glob Public Health. 2023 Oct 26;3(10):e0001978. doi: 10.1371/journal.pgph.0001978 (PMC10602344; doi:10.1371/journal.pgph.0001978)
Supplement: S2 File — (PDF) [file pgph.0001978.s004.pdf]

IRB APPROVAL OF APPLICATION

March 2, 2021

Dear Elizabeth K Harrington:

On 3/2/2021, University of Washington IRB Committee B reviewed the following application:

Type of Review:	Initial Study
Title of Study:	Reproductive empowerment and contraceptive choice among adolescent girls and young women in Kenya: A person-centered approach to unintended pregnancy prevention (Phase 1)
Investigator:	Elizabeth K Harrington
IRB ID:	STUDY00011772
Funding:	Name: National Institute of Child Health and Human Development (NICHD), Grant Office ID: A149870, Funding Source ID: K12HD001264-21 Funding Title(s): "Women's Reproductive Health Research Career Development Program"
IND, IDE, or HDE:	None

IRB Approval

Under FWA #00006878, the IRB approved your activity.

- **Depending on the nature of your study, you may need to obtain other approvals or permissions to conduct your research. For example, you might need to apply for access to data or specimens (e.g., to obtain UW student data). Or, you might need to obtain permission from facilities managers to approach possible subjects or conduct research procedures in the facilities (e.g., Seattle School District; the Harborview Emergency Department).**
- COVID NOTE: Researchers must comply with current infection control requirements and complete a self-assessment that activities fit within allowable research as described on the HSD website.
- Your application qualified for expedited review ("minimal risk"; Category 7).
- Under the Revised Common Rule this IRB approval is valid until study completion. In other words, there is no expiration date and you are not required to submit Continuing Review Reports to maintain your approval. However, you are still required to (1) obtain IRB approval before making any changes (modifications) to your research, and (2) provide the IRB with any Reportable New Information such as breaches of confidentiality or unanticipated problems.
- This approval applies only to the activities described in your application (including any references to specific grant sections). It does not include other activities that may be described in your grant or contract.

- Your study automatically has a Certificate of Confidentiality (CoC), because you have NIH funding. A description of the CoC protections and responsibilities has been placed in your study's Documents section.
- If you plan to continue data collection past the expiration of your NIH funding and the CoC, contact the Human Subjects Division prior to the end of your funding. We will help you determine whether you need to apply for a CoC extension.

Determinations, waivers, and regulations

The IRB made the determinations and waivers listed in the table below. Note that any granted waivers of consent or parent permission do not override a subject's refusal to provide broad consent.

Requirement	Determination or Waiver
Required elements of consent	Screening Questionnaire - Waived: risks, benefits, research contact, HSD contact, research-related injury, no penalty or loss of benefits, discontinuation
Documentation of consent	Waived for screening questionnaire during recruitment
Involvement of children	Approved
Parental permission	Waived
Assent	Assent is required

Location of documents

Use the consent, parental permission, and assent forms that were approved and stamped by the IRB. They can be downloaded from the Final column under the **Documents tab** in Zipline.

In addition, HSD has uploaded the following documents to the **Documents tab** in Zipline:

- Certificate of Confidentiality Acknowledgement Letter

Thank you for your commitment to ethical and responsible research. We wish you great success!

Sincerely,

Jordyn Wheeler
IRB Administrator | Human Subjects Division | University of Washington
Jwl44@uw.edu | 206.543.2529
