# Supplementary material for: Adaptation of the sexual and reproductive empowerment scale for adolescents and young adults in Kenya
Source: PLOS Glob Public Health. 2023 Oct 26;3(10):e0001978. doi: 10.1371/journal.pgph.0001978 (PMC10602344; doi:10.1371/journal.pgph.0001978)
Supplement: S3 File — (PDF) [file pgph.0001978.s005.pdf]

KENYA MEDICAL RESEARCH INSTITUTE

P.O. Box 54840-00200, NAIROBI, Kenya
Tel: (254) 2722541, 2713349, 0722-205901,0733-400003, Fax: (254) (020) 2720030
Email: director@kemri.org, info@kemri.org, Website. www.kemri.org

KEMRI/RES/7/3/1 **May 17, 2021**

TO: **PROF. ELIZABETH K. HARRINGTON AND PROF. ELIZABETH BUKUSI**
PRINCIPAL INVESTIGATORS

THROUGH: **THE DEPUTY DIRECTOR, CMR**
NAIROBI

Dear PIs,

RE: KEMRI/SERU/CMR/P00152/4193 *(RESUBMISSION OF INITIAL SUBMISSION)*: REPRODUCTIVE EMPOWERMENT AND CONTRACEPTIVE CHOICE AMONG ADOLESCENT GIRLS AND YOUNG WOMEN IN KENYA: A PERSON-CENTERED APPROACH TO UNINTENDED PREGNANCY PREVENTION (*VERSION 1.2 DATED 3 MAY 2021*)

Reference is made to your letter dated May 3, 2021. The KEMRI Scientific and Ethics Review Unit (SERU) acknowledges receipt of the revised study documents on May 7, 2021.
The Committee received and reviewed the following documents:

1. Protocol version 1.2 dated 3 May 2021
2. MARA Response Cover Letter dated 3 May 2021
3. SERU Letter Dated 29th April 2021

This is to inform you that the Committee notes that the following issues raised during the 310th Committee B meeting of the KEMRI Scientific and Ethics Review Unit (SERU) held on **April 21, 2021**, have been adequately addressed.

Consequently, the study is granted approval for implementation effective this day, **May 17, 2021** through to **May 16, 2022**. Please note that authorization to conduct this study will automatically expire on **May 16, 2022**. If you plan to continue with data collection or analysis beyond this date, please submit an application for continuation approval to SERU by **April 4, 2022**.

Please note that only approved documents including (informed consents, study instruments, Material Transfer Agreement) will be used. You are required to submit any proposed changes to this study to SERU for review and the changes should not be initiated until written approval from SERU is received. Any unanticipated problems resulting from the implementation of this study should be brought to the attention of SERU and you should advise SERU when the study is completed or discontinued.

CONFIDENTIAL

Prior to commencing your study, you will be expected to obtain a research license from National Commission for Science, Technology and Innovation (NACOSTI) https://oris.nacosti.go.ke and also obtain other clearances needed.

Yours faithfully,

ENOCK KEBENEI,
THE ACTING HEAD,
KEMRI SCIENTIFIC AND ETHICS REVIEW UNIT.
