# Supplementary material for: Adaptation of the sexual and reproductive empowerment scale for adolescents and young adults in Kenya
Source: PLOS Glob Public Health. 2023 Oct 26;3(10):e0001978. doi: 10.1371/journal.pgph.0001978 (PMC10602344; doi:10.1371/journal.pgph.0001978)
Supplement: S5 File — (PDF) [file pgph.0001978.s007.pdf]

Structured Reflexivity Statement[1]

Study conceptualization	1. How does this study address local research and policy priorities?<br><br>**Every study that is reviewed by the Kenya Medical Research Institute (KEMRI) Centre Scientific Steering Committee and Scientific Ethics Research Unit must address Kenyan research and policy priorities and the Sustainable Development Goals. This study's *research programme area* was "sexual, reproductive, adolescent, and child health." The *key performance area* was "health research and innovation," and the KEMRI *strategy addressed* was two-fold: "conduct research for human health in priority areas, and strengthen innovations in research for human health." This research is relevant to Sustainable Development Goal 3 (good health and wellbeing) and 5 (gender equality).**<br><br>2. How were local researchers involved in study design?<br><br>**Dr. Harrington and Prof. Bukusi worked collaboratively on study design. Dr. Upadhyay provided mentorship in scale adaptation and development to Dr. Harrington.**
Research management	1. How has funding been used to support the local research team(s)?<br><br>**This study was funded by a K12 Career Development Award from the National Institutes of Health. Approximately 80% of the funding over two years was subcontracted to KEMRI for study team salaries and research materials.**
Data acquisition and analysis	1. How are research staff who conducted data collection acknowledged?<br><br>**Annabell Dollah conducted the majority of data collection; she is listed as a co-author. Caroline Omom also conducted data collection and is recognized in the Acknowledgments section of the manuscript.**<br><br>2. How have members of the research partnership been provided with access to study data?<br><br>**KEMRI and the University of Washington have a data sharing agreement with joint access and ownership of study data.**<br><br>3. How were data used to develop analytical skills within the partnership?<br><br>**Two early career KEMRI researchers who had content expertise and a strong interest in qualitative data analysis were trained in qualitative coding and analysis as part of this research. Our analytic team met**

[1] Morton B, Vercueil A, Masekela R, Heinz E, Reimer L, Saleh S, et al. Consensus statement on measures to promote equitable authorship in the publication of research from international partnerships. Anaesthesia. 2022;77(3).

	**weekly for over a year to both rigorously analyze the data and provide mentorship to early career researchers.**
Data interpretation	1. How have research partners collaborated in interpreting study data?<br><br>**Qualitative data analysis is equivalent to interpreting the data. Please see above, Data acquisition and analysis, question 3.**
Drafting and revising for intellectual content	1. How were research partners supported to develop writing skills?<br><br>**Early career KEMRI researchers each contributed 2-3 analytic memos, which were approximately 2-page summaries of key themes that intersperse participant quotations and analytic text contextualizing the quotations. These memos became the thematic basis of part of the results section. All co-authors participated in revising the draft manuscript written by Dr. Harrington.**<br><br>2. How will research products be shared to address local needs?<br><br>**Our dissemination plan states that we will share the data in the form of a report after the quantitative component of the study has been completed and results of the psychometric analysis of the adapted SRE scale are available.**
Authorship	1. How is the leadership, contribution and ownership of this work by LMIC researchers recognised within the authorship?<br>2. How have early career researchers across the partnership been included within the authorship team?<br><br>How has gender balance been addressed within the authorship?
Training	1. How has the project contributed to training of LMIC researchers?<br><br>**As in data acquisition and analysis, LMIC researchers engaged in training and mentorship around qualitative methods, including in-depth interviews and cognitive interviews, and qualitative data analysis.**
Infrastructure	1. How has the project contributed to improvements in local infrastructure?<br><br>**The project has not contributed to improvements in local infrastructure.**
Governance	1. What safeguarding procedures were used to protect local study participants and researchers?<br><br>**This project applied the highest ethical standards to our work, which engaged adolescents and young women, some of whom were minors. Our research was reviewed and approved by institutional review boards in the United States and Kenya, and we also received a Research Permit. We used a variety of safeguards for study participants, including research staff experience, research staff training, private inclusion criteria, rigorous written consent and assent processes, strong data management and security to prevent breaches of confidentiality, and referral processes for participants experiencing abuse.**
